# Tincture of Iodine in Infected Wounds

**Published:** 1901-08-17

**Authors:** 


### TINCTURE OF IODINE IN INFECTED WOUNDS.

Dr. Carl Beck,[1] of New York, draws attention to the fact that while the treatment of wounds made by the surgeons has nowadays reached a state of perfection which might nearly be called ideal, it is yet practically impossible thoroughly to disinfect a wound, in the common sense of the word, after infection has once occurred. Some, indeed, have gone so far as to deny the possibility of disinfection even though an antiseptic fluid be applied immediately after infection. The experiments of Schimmelbusch, consisting in the inoculation of anthrax bacilli at the distal end of a mouse's tail, are well known. No matter what the kind and strength of the disinfecting agent used immediately after inoculation, mice so treated died of sepsis except when the tail was amputated at most five minutes after infection. When there has been risk of infection one must take care to minimise as far as possible the supply of the nutrient materials in which infective organisms most easily grow by employing broad incisions, by the removal of mortifying and necrosed tissues, and by absorbing the wound secretion by gauze and especially by iodoform gauze. But in addition to this it would evidently be a great advantage could we soak the tissues which have been exposed to infection with some disinfectant which would penetrate them for some depth and thus might at any rate hold in check the growth of the infecting organisms. Such a disinfectant Dr. Carl Beck thinks we possess in tincture of iodine, which, though originally used only for the disinfection of the skin, possesses a certain permeating power. He has for some time employed it methodically in all infected wounds, and practically all wounds which are not inflicted by an aseptic surgeon on an aseptic field must be regarded as infected. The tincture has been liberally applied once over the carefully dried wound surface, and fifteen minutes afterwards examination has shown that there was evidence of permeation, and that no cultures could be obtained from such areas. No general disturbance has been observed in the large number of patients in whom the iodine treatment has been tried, although in two cases iodine reaction was found in the urine three and four hours after the application. The further treatment of the cases was carried out on general principles, and all of them ran a favourable course.

[1] New York Medical Record, Aug. 3rd.

